# Increased Prevalence of *Trichinella* spp., Northeastern Germany, 2008

**DOI:** 10.3201/eid1606.091629

**Published:** 2010-06

**Authors:** Gunter Pannwitz, Anne Mayer-Scholl, Aleksandra Balicka-Ramisz, Karsten Nöckler

**Affiliations:** Veterinär-und Lebensmittelüberwachungsamt Ostvorpommern, Anklam, Germany (G. Pannwitz); Federal Institute for Risk Assessment, Berlin, Germany (A. Mayer-Scholl, K. Nöckler); West Pomeranian University, Szczecin, Poland (A. Balicka-Ramisz); 1 These authors contributed equally to this article.

**Keywords:** Trichinella spp., Germany, Poland, wild boars, raccoon dogs, foxes, prevalence, outbreak, parasites, research

## Abstract

In 2008, a *Trichinella* spp. outbreak occurred on a small family-owned pig farm in Mecklenburg–Western Pomerania in northeastern Germany. To obtain epidemiologic information on this outbreak, we determined that after 2005 the prevalence of *Trichinella* spp. in wild boars has increased in this region of Germany. We discuss the potential role of the raccoon dog in the increase in *Trichinella* spp. prevalence in the sylvatic cycle in this region. We believe that this increase could pose a threat to pigs kept in back yard conditions, and we provide recommendations to ensure public health safety.

## Abstract 2

Migration of raccoon dogs from Poland may have caused this change.

Nematodes of the genus *Trichinella* infect a broad variety of mammals, birds, and reptiles and are distributed worldwide (*1*). Trichinellosis is a foodborne zoonotic disease caused by a parasite. Human infections occur after ingestion of raw or inadequately cooked meat containing parasite larvae (*2*). Pigs represent a major source of human infection, but meat from horses, wild boars, bears, and badgers have also played a major role during outbreaks (*3*,*4*).

*Trichinella* spp. can be transmitted by domestic and sylvatic cycles. The domestic cycle is maintained by feeding of swill to pigs and pigs feeding on animal carcasses or on synanthropic animals (e.g., rats, mice). In Germany, the domestic cycle disappeared >30 years ago (*5*). During 1998–2007, ≈436 million pigs were slaughtered and tested for *Trichinella* spp. by using artificial digestion according to regulation (European Commission [EC]) no. 2075/2005 (*6*). However, in 2003, 1 positive case was reported; it was in a pig kept in a back yard (*7*).

Currently, the major *Trichinella* spp. reservoir and source of infection for domestic pigs in Germany is wild boars (*Sus scrofa*), raccoon dogs (*Nyctereutes procyonoides*), and foxes (*Vulpes vulpes*). The most prevalent *Trichinella* spp. is *T. spiralis*, followed by *T. britovi* and *T. pseudospiralis* (*8*).

In the past 30 years, sporadic human infections with *Trichinella* spp. (0–50 reported cases per year) have occurred in Germany (*7*,*9*). These infections are usually linked to consumption of contaminated meat during holiday visits to high-risk countries (*10*). Autochthonous outbreaks occur infrequently, such as during 2005–2006, when 17 members of a large family in Mecklenburg–Western Pomerania were infected with *Trichinella* spp. after consumption of meat products from a pig reared and slaughtered at home (*11*).

We report a *Trichinella* spp. outbreak on a small family-owned pig farm in Mecklenburg–Western Pomerania in northeastern Germany during December 2008. We show that after 2005, the prevalence of *Trichinella* spp. in wild boars has increased in this region of Germany. Furthermore, we discuss the possibility that increased *Trichinella* spp. prevalence in wild boars is the result of high prevalence of the disease in neighboring Poland. The potential role of migration of raccoon dogs from Poland into Germany is also considered as a factor of increased prevalence.

## Materials and Methods

### Outbreak Investigations

Veterinary inspections of the outbreak farm were performed with emphasis on production type, location, potential contacts with wild animals (including rodents), feeding habits, *Trichinella* spp. status of animals on origin farms, and human factors. Meat and blood samples of 5 slaughtered pigs from the outbreak farm were sent to the National Reference Laboratory for Trichinellosis at the Federal Institute for Risk Assessment in Berlin, Germany.

### Laboratory Examinations

#### Muscle Samples

All muscle samples from Germany and Poland were examined by artificial digestion (magnetic stirrer method) according to regulation (EC) no. 2075/2005 (*6*). A mouse caught in a trap on the outbreak farm was examined by using the same method.

#### PCR

Larvae were isolated from muscle tissues and washed 4× with distilled water on ice and stored in 5 µL of distilled water at –20°C. DNA extraction and PCR were performed as described by Pozio and La Rosa (*12*).

#### Serologic Testing of Blood and Meat Juice

Venous blood was collected from live pigs. Meat was obtained from the diaphragm pillar of slaughtered pigs, cut in small pieces, put in plastic bags, and frozen at –20°C for 3 days. After thawing, the meat was squeezed and meat juice was collected and stored at –20°C. Serum samples were examined by using an in-house ELISA as described by Nöckler et al. (*13*). This assay is based on the excretory–secretory antigen (*14*).

### Sampling, Data Management, and Statistical Analysis

There are currently no reliable estimates of the number of wild boars in Germany. However, the number of wild boars hunted during a hunting year in a specific region is considered proportional to the size of the wild boar population (*15*). To optimally control the wild boar population and prevent damage to land or crops and the spread of disease, wild boars in Germany are hunted annually (*16*). Depending on climate, reproduction rate, and hunting success, ≈50%–100% of the animals born in a given year are hunted (killed) in Germany each year (*17*). According to specifications of German hunting associations, the number of wild boars hunted should consist of 80% piglets (maximum age 1 year), 10% juveniles (1–2 years of age), and 10% older sows. However, because hunters generally focus on older piglets and juveniles, the suggested number of wild boars hunted is not representative of the population and is biased toward younger animals. This factor could lead to an underestimation of the *Trichinella* spp. prevalence in wild boars because young animals only have a short time span during which they can become infected.

The number of foxes and raccoon dogs hunted is also thought to reflect changes in the sizes of the populations of these species. Raccoon dogs and foxes are not examined for *Trichinella* spp. according to regulation (EC) no. 2075/2005, and currently, Germany has no countrywide monitoring program for *Trichinella* spp. in these animals. Animals examined were part of research initiatives. Therefore, sampling of raccoon dogs and foxes is less representative than that of wild boars. Information on wild boars examined each year for *Trichinella* spp. in the German Federal States during 2002–2008 was supplied by the German Federal Statistics Office (*18*).

During 2002–2008, prevalences of *Trichinella* spp.–positive wild boars in Ostvorpommern, Germany, were compared with those in Mecklenburg–Western Pomerania and in the remaining German Federal States. Data for *Trichinella* spp. prevalences in foxes and raccoon dogs hunted in Mecklenburg–Western Pomerania during 1993–2008 and in wild boars and foxes hunted during 2004–2008 on Island Wolin, Poland, which borders Ostvorpommern, were also analyzed. We determined 95% confidence intervals from the binomial distribution as described by Clopper and Pearson (*19*). Descriptive statistics were calculated by using available online resources (www.statpages.org).

## Results

### Outbreak Investigations

In November 2008, an 11-month-old pig (German Landrace × Large White) was declared positive for *Trichinella* spp. at an abattoir in Ostvorpommern, a district in Mecklenburg–Western Pomerania. The day after detection of *Trichinella* larvae, veterinarians of the Veterinary and Food Safety Authority visited the outbreak farm and conducted epidemiologic investigations. A blood sample obtained for swine fever monitoring shortly before slaughtering was sent to the National Reference Laboratory for Trichinellosis. The remaining 4 pigs at this site were slaughtered ≈2 weeks after the first case of *Trichinella* infection was identified.

#### Description of Farm

The farm was a small holding of ≈0.5 acres at the edge of a village bordering a large field. In addition to the 5 pigs, sheep, poultry, cats, and a dog also lived on the farm. The pigs were penned in an old, closed building and allowed to enter a small outdoor yard once a week. The farm was run in accordance with the German Pig-Keeping Hygiene Ordinance.

#### Rodent Manifestations

During the summer of 2008, the farmer observed some rats on the premises. A professional pest control company was contacted after the slaughtering of all pigs, but no rodent infestation was detected.

#### Feeding

The pigs were routinely fed steamed potatoes, potato ensilage, fodder beets, feed grain, sugar beet pellets, and water. According to the farmer, the pigs were not fed swill.

#### *Trichinella* spp. Status of Origin Farm

At the beginning of 2008, a total of 3 *Trichinella* spp.–positive animals had been bought as weaned pigs from a small breeding farm, which had 8 breeding sows, 2 boars, and ≈60 piglets ≈3 km from the outbreak farm. Blood samples were taken from 16 animals (including 8 breeding sows) for testing by ELISA. All pigs sold from and slaughtered at this breeding farm in 2008 were traced and confirmed to have been negative for *Trichinella* spp.

The 2 *Trichinella* spp.–negative pigs from the outbreak farm were obtained as finishers from a large commercial fattening unit that delivered hundreds of finished pigs per week to an EU-licensed abattoir. To date, all pigs from the commercial piggery have been negative for *Trichinella* spp.

#### Human Factors

Until the fall of 2008, a family member living on the outbreak farm was employed as a cook in a restaurant near the border with Poland. The restaurant is well known for game dishes and has its own meat-cutting room and area for selling meat. During an inspection of the restaurant, veterinarians found evidence that meat offal was being supplied to unknown owners of animals. However, additional information (number or identity of owners of animals) could not be obtained.

### Laboratory Examinations

#### Muscle Examination

One *Trichinella* spp.–positive pig slaughtered had 299 larvae/g of tissue, and the 2 other pigs kept in the area had 1.2 and 1.3 larvae/g of tissue, respectively (Table 1). The other 2 pigs kept in a separate area on the farm were negative for *Trichinella* spp. Larvae from all 3 *Trichinella* spp.–positive pigs were examined by PCR and identified as *T. spiralis*. All 3 *Trichinella* spp.–positive carcasses were classified as unfit for human consumption.

**Table 1:** Results of laboratory examinations of 5 pigs for *Trichinella* spp. on outbreak farm, Germany, 2008*

Animal no.	Diaphragm pillar			IgG ELISA (meat juice)			IgG ELISA (serum)	
	Artificial digestion	Larvae/g		Titer	Result		Titer	Result
1	+	299		>128	+		>1,280	+
2	+	1,2		32	+		320	+
3	+	1,3		64	+		320	+
4	–	0		<10	–		10	–
5	–	0		<10	–		<10	–

*Ig, immunoglobulin.

#### Serologic Examination

ELISAs were conducted for blood and meat juice samples from the 5 pigs on the outbreak farm. Results were consistent with those of the magnetic stirrer method (Table 1). All 16 blood samples from the 3 *Trichinella* spp.–positive pigs from the origin farm were negative for *Trichinella* spp. by ELISA. Antibodies against *Trichinella* spp. were not detected in the serum of the farmer (immunoglobulin G titer <10, immunoglobulin M titer <40).

### Prevalence of *Trichinella* spp. in Wild Boars in Germany, 2002–2008

In Germany, examination of wild boar carcasses intended for human consumption is compulsory (*6*). Because reporting to the German Federal Statistical Office is obligatory, the number of wild boars examined per year was considered a representative sampling unit.

**Figure 1:**
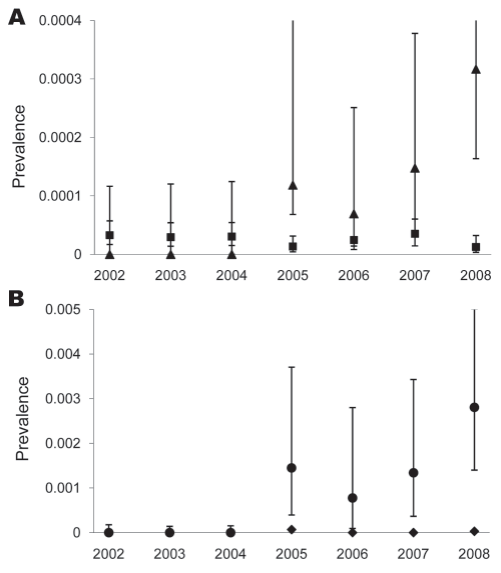
Yearly prevalence of *Trichinella* spp. in wild boars in A) Mecklenburg–Western Pomerania and B) Ostvorpommern, Germany. Nonoverlapping error bars indicate significance at p<0.05. Squares, Germany other than Mecklenburg–Western Pomerania; triangles, Mecklenburg–Western Pomerania; circles, Ostvorpommern; diamonds, Mecklenburg–Western Pomerania.

The yearly prevalence of *Trichinella* spp. in wild boars in Germany during 2002–2008 ranged from 0.0027% to 0.0032% (Table 2). In 2005, there was a sudden increase in *Trichinella* spp. prevalence in wild boars in Mecklenburg–Western Pomerania compared with the rest of Germany. During 2005 and 2008 (Figure 1, panel A) and during 2005–2008, more *Trichinella* spp.–positive wild boars were detected in Mecklenburg–Western Pomerania than in the rest of Germany.

**Table 2:** Western blot results for prevalence of *Trichinella* spp. in wild boars, Mecklenburg– Western Pomerania and Ostvorpommern, Germany

Year	Germany, no. positive/no. tested (%)	Mecklenburg–Western Pomerania, no. positive/no. tested (%)	Ostvorpommern District, no. positive/ no. tested (%)
2002	12/397,425 (0.0032)	0/31,667 (0)	0/3,616 (0)
2003	10/370,187 (0.0027)	0/30,632 (0)	0/3,201 (0)
2004	11/ 390570 (0.0028)	0/29,592 (0)	0/2,992 (0)
2005	11/402,996 (0.0027)	6/32,227 (0.0186)	4/2,760 (0.1449)
2006	8/272,258 (0.0029)	2/28,764 (0.0070)	2/2,577 (0.0776)
2007	9/282,442 (0.0032)	4/27094 (0.0148)	4/2,983 (0.1341)
2008	16/354,118 (0.0045)	12/37,880 (0.0317)	11/3,921 (0.2805)

Within Mecklenburg–Western Pomerania, 21 (87.5%) of 24 *Trichinella* spp.–positive results were in wild boars in Ostvorpommern, the district where the outbreak farm was located. *Trichinella* spp. prevalence in Oatvorpommern was higher in 2005, 2007, and 2008 than in the rest of Mecklenburg–Western Pomerania (Figure 1, panel B).

For verification and species identification, 20 *Trichinella* spp.–positive wild boar samples obtained during 2005–2008 were sent to the National Reference Center for Trichinellosis. A total of 80% (14) were identified as *T. spiralis*, 15% (3) as *T. pseudospiralis*, and 1 as a mixed infection (*T. spiralis* and *T. pseudospiralis*) (*20*). Larval load ranged from 2 to 922 larvae/g of tissue.

### *Trichinella* spp.–Positive Raccoon Dogs and Foxes, Mecklenburg–Western Pomerania

The number of raccoon dogs and red foxes hunted in Mecklenburg–Western Pomerania is shown in Table 3. During 1993–2008, a sharp increase in raccoon dogs hunted was observed; the number of foxes hunted remained constant over this period. No difference was found between the number of foxes and raccoon dogs hunted in Ostvorpommern and those hunted in more westward districts of similar sizes in Mecklenburg–Western Pomerania.

**Table 3:** Number of raccoon dogs and red foxes hunted, Mecklenburg–Western Pomerania, Germany

Years	Raccoon dogs	Red foxes
1993–1996	541	123,316
1997–2000	9,324	144,260
2001–2004	43,259	122,820
2005–2008	78,311	116,322

In Germany, raccoon dogs and foxes are not routinely examined for *Trichinella* spp. In a monitoring program conducted in Mecklenburg–Western Pomerania during February 2006–January 2007, a total of 100 raccoon dogs and foxes were examined by using the magnetic stirrer method. *Trichinella* spp. prevalence was 4.0% in raccoon dogs and 1.0% in foxes. In a smaller district-level monitoring study during February–August 2006, a total of 3 of 46 raccoon dogs from Ostvorpommern and a neighboring district were positive for *Trichinella* spp. (prevalence 6.5%). Larval load ranged from 0.06 to 65 larvae/g of tissue. Four of 7 raccoon dogs were infected with *T. spiralis* and 2 with *T. pseudospiralis*. One raccoon dog from Ostvorpommern had a mixed infection (*T. spiralis* and *T. pseudospiralis*).

### *Trichinella* spp. Prevalence in Wild Boars and Raccoon Dogs, Ostvorpommern

**Figure 2:**
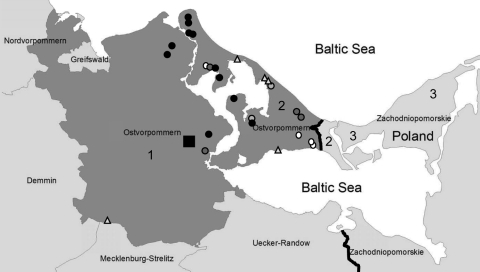
Region in Germany and Poland investigated for *Trichinella* spp. 1, Ostvorpommern, Germany; 2, Usedom Island, Germany; 3, Wolin Island, Poland. Triangles, *Trichinella* spp.–positive cases in raccoon dogs; circles, cases in wild boars; square, location of outbreak farm; white circles and triangles, cases in 2005; light gray circles, cases in 2006; dark gray circles, cases in 2007; black circles, cases in 2008.

Ostvorpommern is located in the eastern part of Mecklenburg–Western Pomerania, Germany (Figure 2). Part of this district is on Usedom Island, an island in the Baltic Sea. The western part of Usedom Island is in Germany and the eastern part is in Poland. Of the 26 reported *Trichinella* spp. infections in wild animals (21 in wild boars and 5 in raccoon dogs) in Ostvorpommern during 2005–2008, a total of 80.7% were found on Usedom Island (Figure 2).

### *Trichinella* spp. Prevalence in Wild Boars and Foxes, Wolin Island, Poland, 2004–2008

On Wolin Island, which borders Usedom Island, 22 (3.27%) of 672 wild boars examined during 2004–2008 were positive for *Trichinella* spp. During the same period, 6 (4.22%) of 142 foxes were positive for *Trichinella* spp. (Table 4).

**Table 4:** Western blot results for *Trichinella* spp. in wild boars and red foxes, Wolin Island, Poland

Year	Wild boars, no. positive/ no. tested (%)	Red foxes, no. positive/ no. tested (%)
2004	1/107 (0.93)	0/21 (0)
2005	1/150 (0.67)	2/32 (6.25)
2006	2/84 (2.38)	1/26 (3.85)
2007	3/86 (3.490	1/25 (4.00)
2008	15/245 (6.12)	2/38 (5.26)

## Discussion

In December 2008 a *Trichinella* spp. outbreak occurred on a pig farm in Mecklenburg–Western Pomerania, Germany. Although the affected animals originated from a farm with similar biosecurity levels as the outbreak farm, negative serologic results for pigs at the origin farm and the longer period spent on the outbreak farm make infection on the outbreak farm more likely.

Potential infection sources were manifold. Although trichinellosis is not a contagious disease, all pigs kept in 1 area were positive for *Trichinella* spp. This finding suggests a common source of infection. At the time of outbreak investigations in December 2008, no evidence of rodents was found on the farm. However, the role of rats as a *Trichinella* spp. maintenance host is unclear, and infection in rats is considered a marker for infection in pigs (*21*).

Feeding of infectious meat (e.g., wild boar) can also lead to *Trichinella* spp. infection in pigs. The farmer on the outbreak farm had access to game meat because a family member worked at a restaurant in which game meat offal was given to owners of animals. Although the direct circumstances could not be elucidated, game meat offal seems to be the most likely source of infection.

The yearly prevalence of *Trichinella* spp. in wild boars in Germany ranged from 0.0027% in 2002 to 0.0045% in 2008. In contrast, during 1975–2005, *Trichinella* spp. were not detected in wild boars in Mecklenburg–Western Pomerania. Since 2005, *Trichinella* spp. prevalence has increased in Mecklenburg–Western Pomerania in wild boars in comparison with the rest of Germany. Mecklenburg–Western Pomerania, the district in which Ostvorpommern is located, has been predominantly affected. A total of 80.7% of all wild boars and raccoon dogs positive for *Trichinella* spp. were found on Usedom Island.

Since 2005, the trichinoscopic method for larvae detection has been replaced by more sensitive methods of artificial digestion (*6*). Because new legislation regarding trichinellosis was implemented at the same time in Germany, it is unlikely that higher *Trichinella* spp. prevalence in wild boars in Mecklenburg–Western Pomerania can be attributed to the use of this improved diagnostic technique.

Human trichinellosis is regularly reported in Poland. Thirty-five human outbreaks and 702 cases were reported in Poland during 2002–2007 (*22*). Because of stringent control measures in recent years, the primary source of human infection has changed from pork to wild boar meat (*23*). During 1999–2004, Balicka-Ramisz et al. (*24*) examined >56,000 wild boars in West Pomerania, a province in northwestern Poland bordering Mecklenburg–Western Pomerania, Germany. *Trichinella* spp. prevalence in wild boars in this region increased 8-fold from 0.12% to 1.48% during this period. In the same study, >500 foxes from West Pomerania were tested; *Trichinella* spp. prevalence was 4.4%. *Trichinella* spp. prevalence in wild boars on Wolin Island Poland was even higher (6.12% in 2008).

In Germany, the *Trichinella* spp. prevalence in foxes is <1% (*25*–*27*). However, the raccoon dog can also play a major role in maintenance of the *Trichinella* spp. sylvatic cycle (*28*). Raccoon dogs were first reported in Poland in 1955 (*29*); this species has become established in eastern Europe and has extended its settlement area to the west and south. The raccoon dog population in Germany has increased dramatically in recent years. In the former German Democratic Republic, only 58 raccoon dogs were hunted before 1987 (*30*). In 2001–2002, a total of ≈12,000 raccoon dogs were hunted in Germany; >96.0% were hunted in Mecklenburg–Western Pomerania and Brandenburg, the most eastern German Federal States (*31*). Studies in eastern Germany showed that 25.9%–35.1% of the raccoon dog diet is carrion (*32*). When intensity of infection in these animals was compared with that of other carnivores, the raccoon dog had higher larvae loads (*33*,*34*), which indicates the role of this species as a *Trichinella* spp. reservoir. In addition, *T. spiralis* can survive in rat carcasses during the summer in northern Europe for 2–4 weeks (*33*). Thiess et al. (*28*) examined 120 raccoon dogs from the northern part of Brandenburg; *T. spiralis* was identified in 5.0%. Results of that study support our findings in Mecklenburg–Western Pomerania.

Our data are insufficient to fully ascertain risk factors specific for the increase in *Trichinella* spp. prevalence in this region. Although for some years the raccoon dog has become widespread in Mecklenburg–Western Pomerania and Brandenburg in recent years, the *Trichinella* spp. prevalence in wild boars has increased mainly on Usedom Island. As for wild boars, the number of raccoon dogs hunted is considered to be proportional to the size of the total population. During 2005–2008, ≈34,000 raccoon dogs (1.15 raccoon dogs/km^2^) were hunted in Brandenburg. During the same period, 3.38 raccoon dogs/km^2^ were hunted in Mecklenburg–Western Pomerania, which indicated that the raccoon dog population in this region is larger than that in Brandenburg (*35*–*38*). During this time, numbers of wild boars and foxes did not differ between these 2 regions of Germany, and the hunting activity was expected to be similar in both regions.

The optimal habitat for raccoon dogs is wet areas and fields, small forests, and large ditches bordered by thick vegetation (*39*); this habitat corresponds to the landscape in northeastern Germany and northwestern Poland. Also, since 1960, large areas of the Wolin Island have been designated as a national park, which may have led to an increase in the raccoon dog population. Because the body of water separating Wolin Island from Usedom Island is narrower than the Stettiner Haff Lagoon and the Untere Oder (part of the Oder River) to the south, raccoon dog migration from east to west might have occurred through this route. Whether future increased spread of raccoon dogs in Brandenburg will result in increased *Trichinella* spp. prevalence in wild boars is unclear.

In summary, since 2005, an increase in *Trichinella* prevalence spp. in wild boars has occurred in Mecklenburg–Western Pomerania, Germany. This increase in *Trichinella* spp. prevalence in the sylvatic cycle may be associated with spread of raccoon dogs in this region. This increase poses a threat to public health because of pigs kept in backyards. Backyard farming is fairly common in the region and because of economic reasons has been traditionally associated with feeding of kitchen scraps to pigs.

For intensive pig production units that practice high levels of biosecurity, risk for *Trichinella* spp. infection is low. Although feeding with swill is illegal (*40*), feeding of kitchen scraps including wild boar offal still occurs, which increases risk for human outbreaks. Although scavenging of wild boar, fox, or raccoon dog carcasses by pigs is rare, exposure of domestic pigs kept outdoors to *Trichinella* spp. cannot be excluded. Therefore, farmers should be made aware of increased potential risk factors, especially feeding with swill. Also, hunters should be encouraged to remove carcasses from the forest or bury them at an appropriate depth. It would also be advisable to monitor whether the western and southern migration of raccoon dogs is indicative of increased *Trichinella* spp. prevalence in the sylvatic cycle in newly settled areas. Furthermore, programs are needed that emphasize the necessity of ensuring testing for *Trichinella* spp. infection in all wild boars intended for human consumption and promoting education of humans regarding thorough cooking of meat to guarantee food safety.
